# The Role of Decompressive Craniectomy in Limited Resource Environments

**DOI:** 10.3389/fneur.2019.00112

**Published:** 2019-02-26

**Authors:** Angélica Clavijo, Ahsan A. Khan, Juliana Mendoza, Jorge H. Montenegro, Erica D. Johnson, Amos O. Adeleye, Andrés M. Rubiano

**Affiliations:** ^1^INUB/MEDITECH Research Group, El Bosque University, Bogotá, Colombia; ^2^MEDITECH Foundation, Clinical Research, Cali, Colombia; ^3^Global Health Research Group on Neurotrauma, University of Cambridge, Cambridge, United Kingdom; ^4^Department of Surgery, University of Pittsburgh School of Medicine, Pittsburgh, PA, United States; ^5^College of Medicine, University of Ibadan, and University College Hospital (UCH), Ibadan, Nigeria

**Keywords:** decompressive craniectomy, low- and middle-income countries, brain injury, neurological emergencies, low resources areas

## Abstract

Decompressive craniectomy (DC) is a neurosurgical procedure useful to prevent and manage the impact of high intracranial pressure (ICP) that leads to brain herniation and brain's tissue ischemia. In well-resourced environment this procedure has been proposed as a last tier therapy when ICP is not controlled by medical therapies in the management of different neurosurgical emergencies like traumatic brain injury (TBI), stroke, infectious diseases, hydrocephalus, tumors, etc. The purpose of this narrative review is to discuss the role of DC in areas of low neurosurgical and neurocritical care resources. We performed a literature review with a specific search strategy in web repositories and some local and regional journals from Low and Middle-Income Countries (LMICs). The most common publications include case reports, case series and observational studies describing the benefits of the procedure on different pathologies but with several types of biases due to the absence of robust studies or clinical registries analysis in these kinds of environments.

## Introduction

Decompressive craniectomy (DC) is a neurosurgical procedure where some part of the skull bone is removed for prevent pathological rise in intracranial pressure (ICP), brain herniation and brain tissue ischemia. The procedure improves cerebral hemodynamics and brain oxygenation in patients with high ICP, which could decrease mortality and disability in some cases (1). Several intracranial conditions can generate high ICP, and timely treatment of these emergencies is essential to ameliorate intracranial hypertension that generates hypoxia, ischemia and cerebral herniation (2, 3). In low- and middle-income countries (LMICs) neurosurgical emergency care is sometimes delayed due to the lack of neurosurgical work-force or the absence of formal prehospital systems (4–6). Additionally, an absence of post-operative care infrastructure like intensive care units (ICUs) can generate difficulties in the application of protocols of care, designed in high-income settings (7). As an example, in severe TBI cases secondary to road accidents, the transfer of patients to hospitals with neurosurgical capability can take longer times, delaying specialized management or treatment, creating a natural “selection” process, where more sick patients will die and less severe patients will deteriorate over the next hours (8, 9). Facing this reality, early primary DC has been proposed as a common strategy for bring some “hope” of improvement in patients that arrive in a considerable window of treatment (regularly first 24 h) but will not receive immediately ICU care in the following days after the surgery, because lack of availability. In this opinion article, we will analyze published studies describing the use of DC in the management of neurosurgical emergencies in the context of LMICs.

## Materials and Methods

We performed a wide-range search using specific search terms in PubMed filtering for human studies, 2001 to 2018, case reports, observational studies, clinical reports, clinical studies, guidelines, systematic reviews, randomized control trials, and multi-center studies to identify articles assessing the use of DC for neurotrauma and brain injury in LMICs. Search strategy is available as Supplementary Material. We also performed free-text searches for key words like “decompressive craniotomy” or “low- and middle-income countries” in Google Scholar, DIMDI and some regional journals from Africa, Asia, Latin America, Eastern Europe and South Pacific Region. Filters included English, Portuguese, Spanish and French, from 2001 to 2017. Finally, we reviewed the references of articles identified through this search strategy to identify additional citations for review. We included in this narrative review all human studies assessing the use of DC for neurotrauma or acute brain injury, including stroke, thrombosis, tumor, or infection, in both adult and pediatric patients. For our purposes, decompressive craniectomy encompassed unilateral, bilateral and hemi-craniectomy. We excluded non-human research and articles assessing the use of DC in countries that do not meet World Bank criteria for LMICs, with the exception of Argentina, a high-income country (HIC), as studies from this country contributed to an understanding of regional uses of DC in LMIC regions like Latin America.

## Results

Our search identified more than 2000 citations, including 1,148 studies citations from PubMed. After removing duplicate articles, non-human research, and studies not related to DC or LMICs, we included forty-five studies evaluating the role of DC in neurotrauma and forty-eight additional articles related to the use of the DC in non-traumatic neurosurgical emergencies (articles are described in the Supplementary Material). Of these studies we review only the ones with clear description of methodology, outcome descriptions using validated scales and studies describing the surgical technique.

### Studies From LMICs Related to TBI

We found eight studies from the regions of Latin America, Caribbean and North America discussing the role of DC in TBI. Most of the studies were retrospective, and assessed outcomes such as mortality, survival, and Glasgow Outcome Scale (GOS) or modified Rankin Scale (mRS). Five studies were found from Sub-Saharan Africa, including two from Nigeria, two from South Africa and one from Cameroon. Twelve studies were identified from the East Asia Pacific region. In the Europe & Central Asia region we found three studies from Turkey. From the Middle East, North Africa and South Asian region, we identified 12 studies: three from Pakistan, three from India, three from Afghanistan and Iraq (wartime) and three additional studies from Iran, Jordan and Afghanistan (Supplementary Table 1).

The biggest study from Argentina (10) includes a description of 206 pediatric and adult TBI patients managed with or without DC. Mortality was higher in the patients without DC. A study from Colombia (11), evaluated 106 patients under early hemispheric DC with 66% with GOS 4–5 (moderate deficit and normal neurological status) as outcome after 12 months. Three studies of patients with gunshot wounds to the head, from Mexico, Argentina and Colombia (12–14) evaluated DC as therapeutic options with good survivals between 34 and 74%. The first two studies did not specify the DC technique, but the other used hemispheric and bihemispheric techniques for DC. Studies from Cuba (15, 16) analyzed pediatric patients with bi-frontal and unilateral DC. Nearly 60% of the patients in both studies survived with GOS 4-5.

Observational studies from Sub-Saharan Africa, including samples from Nigeria, South Africa & Cameroon, also reported low mortality rates in patients under hemispheric DC (17–19) or hemispheric and bi-frontal DC (20–22).

Studies from China (23–26) show different results with lower mortality in patients under DC with mixed techniques (unilateral and bi-frontal) in pediatric and adult population. In general survival with GOS 4-5 was over 50%. Other Chinese studies (27, 28) compare early vs. late interventions and small to larger decompressions, finding better outcomes in early and large decompressions.

Studies from Mongolia (29), Malaysia (30), and Thailand (31) also were consistent with benefits of unilateral or bilateral decompressions.

In the Middle East, North Africa and South East Asia regions, several observational studies have been performed in civilian and war settings. A study from Iran (32) with 142 patients and another from India (33) with 1,236 patients treated with unilateral and bilateral decompressions have the largest samples of civilian settings with favorable outcomes in both studies. In the second one, 49% of patients survived with GOS 4-5 at discharge. In Afghanistan and Iraq, studies by military neurosurgeons (34–36), showed the same trend of over 50% survival. In Pakistan and India, other studies have been performed including samples of pediatric and adult patients (37–39). These studies also show survivors with favorable outcome in more than 50% of the patients, using different techniques.

### Studies From LMICs Related to Stroke and Cerebral Venous Sinus Thrombosis (CVST)

We found three studies from Latin American, Caribbean and North American regions (two from Brazil and one from Colombia). Nine studies were identified from the East Asia Pacific region (eight from China and one from Malaysia). Two were found from Europe and Central Asia region (Turkey). Twelve studies were found from Middle East, North Africa and South Asia region: among these, six were from India, two from Iran and one each from Egypt and Pakistan. Among all these studies, patients underwent hemicraniectomy and this operation improved survival as compared to conservative medical therapy. Among survivors, those who underwent surgery had better outcomes and improved quality of life, measured with the modified Rankin Scale (mRS). The DC also was used in studies of CVST: 1 study was found from East Asia Pacific (China), and 5 from Middle East, North Africa and South Asia (four from India and one from Pakistan). All studies showed improved survival rate and favorable outcomes in patients who underwent DC (see Supplementary Tables 1–3).

A study from Brazil (40) presented a cohort of 60 patients with malignant middle cerebral artery (MCA) infarction who underwent unilateral DC. They showed that mortality was higher (67%) in patients > 60 years, while only 44% of the patients from the younger group (<60 years) had mRS 5–6 at 90 days follow-up. A study from China (41) presented data of 219 adult patients in which 31 patients underwent unilateral DC after malignant MCA infarct; they showed higher favorable outcome (32.2%) in patients who underwent DC vs. those who only had medical management (13.3%) at 1-year follow-up. An Indian study (42) showed absolute risk reduction of 45% in mortality at 1-year in the patients who underwent DC vs. medical treatment only in malignant MCA infarct. They found that surgery reduced the odds of moderate to severe disability (mRS 4) by 93.5%. Similarly, another study from India (43) showed 73% survival at 1-year post-surgery, and among the survivors 72% attained the ability to walk independently at this post-surgical milestone. A third study from India (44) presented data of 53 patients; 60% among these were older than 60 years. Their study found that 78% patients aged below 60 years had mRS 0-3 (good outcome) at discharge while only 38% patients aged above 60 years had similar outcome, mRS 0-3, at discharge, demonstrating that DC reduces morbidity and mortality in patients below 60 years. A Malaysian study (45) presented data of 125 patients and among those 90 had DC and 35 received medical treatment. They showed that DC resulted in reduced mortality (30.0 vs. 54.3%) and favorable GOS at discharge. A study from Iran (46) with 60 participants reported reduced mortality and better average GOS (2.93 vs. 1.53) in surgical group vs. medical treatment group. Similarly, they observed better mRS in surgical vs. medical management (3.27 vs. 5.27).

A study from Pakistan (47) showed that DC is beneficial in both dominant and non-dominant side infarctions. In this study the mean surgery time from diagnosis was 60.61 h, which is beyond the recommended period (within 24–48 h). Another study from Iran (48) presented 30 patients with large and deep seated supratentorial intracerebral hemorrhage (ICH) that were randomly divided either in a group where they only received large decompressive hemicraniectomy or in a group where they underwent craniotomy with clot evacuation. They showed that there was no difference in mortality and GOS at 6-months but good outcome (GOS 4-5) was higher (35.3%) in patients who had undergone hematoma evacuation vs. those who had large DC only (30.7%).

A study from Brazil (49) investigated the role of DC in patients with intracranial aneurysms. In their study, they presented 37 cases of DC performed in patients with aneurysms and among them 22 had ruptured aneurysms. In their cohort, 60% survived after DC and they recommended early surgery because it reduced mortality and morbidity.

Six studies from LMICs were found about the role of DC in the management of CVST. A retrospective study with 58 adults from China (50) showed favorable outcomes in 56.9% of the patients who underwent hemicraniectomy for CVST. Similarly, another observational retrospective study from India (51) with 34 adult patients also presented favorable outcomes in 76.4% of patients who underwent unilateral DC for CVST management.

## Studies of DC in Other Pathologies

The procedure also has been applied in management of conditions like infections and tumors; we found 3 case reports regarding the role of DC in infections from Latin American, Caribbean and North American Regions (1 each from Argentina, Brazil and Peru). Two case reports about infection were found from Middle East, North Africa and South Asia region (India). A few other case reports were found regarding the use of DC in malignancies, intracranial demyelinating lesions and vasospasm after subarachnoid hemorrhage (Supplementary Material).

Two case reports from India (52, 53) showed favorable outcomes in patients who underwent hemicraniectomy for the management of Herpes Simplex Encephalitis and Cerebral Toxoplasmosis, respectively. Case reports from Argentina and Mexico (54, 55) showed use of DC in the management of tumors and patients initially improved post-operatively but complications including death and metastasis were observed in long-term follow-up. Another case report of 2 pediatric patients with ICH in acute leukemia from India (56) showed favorable outcomes following DC.

## Discussion

This literature search identified several studies demonstrating the use of decompressive craniectomy in LMICs, for conditions such as traumatic brain injury, stroke, CVST, and other neurosurgical emergencies. Publications assessing the use of decompressive craniectomy for traumatic brain injury in LMICs showed overall favorable outcomes, assessed either as overall survival or GOS on discharge. However, these studies were largely limited to case reports, case series and observational studies from single centers describing the benefits of the procedure on different pathologies. While several studies demonstrated lower mortality or higher GOS in those patients undergoing decompressive craniectomy, these studies were limited by the absence of robust studies or clinical registries analysis in these kinds of environments. In addition, there is substantial variability among the studies with regard to timing and type of decompressive craniectomy performed, as well as in study population (adult or pediatric). While these limitations make it difficult to compare results between studies, it can be seen that decompressive craniectomy is a commonly used procedure for the management of TBI, stroke, and other neurosurgical emergencies in LMICs, and that this procedure has benefits for survival in certain settings.

Recent developments coming specifically from LMICs in the aspects of DC have been described within the brief discussion of the articles included. Due to significant variability in the conditions, procedures, and outcomes assessed we were unable to perform a full systematic review of this topic. However, we conducted a comprehensive search of the literature that identified relevant articles from these environments from many regions of the world.

## Conclusions

Decompressive craniectomy is a frequently used procedure for the management of neurosurgical emergencies in LMIC's according to the available medical literature. The most common publications include case reports, case series and observational studies describing the benefits of the procedure on different pathologies. In most of the observational studies there is a common trend of benefit from the procedure, but the low methodological quality of these studies and a high risk of publication bias does not allow any type of conclusions valid for transferability of knowledge in other regions of the world.

## Author Contributions

AC, AK, JM, JHM, AA, EJ, and AR, contributed equally to the conception, writing and preparation of the manuscript.

### Conflict of Interest Statement

The authors declare that the research was conducted in the absence of any commercial or financial relationships that could be construed as a potential conflict of interest.
