# Supplementary material for: The Role of Decompressive Craniectomy in Limited Resource Environments
Source: Front Neurol. 2019 Feb 26;10:112. doi: 10.3389/fneur.2019.00112 (PMC6399379; doi:10.3389/fneur.2019.00112)
Supplement: Supplementary file 1 [file Data_Sheet_1.pdf]

1. Search Strategy:

"neurology"[MeSH Terms] AND "emergencies"[MeSH Terms] OR (("neurosurgical procedures"[MeSH Terms] OR "neurosurgery"[MeSH Terms]) AND "emergencies"[MeSH Terms] AND "decompressive craniectomy"[MeSH Terms]) OR ("decompressive craniectomy"[MeSH Terms] AND ("income"[MeSH Terms] OR "income"[All Fields]) AND countries [All Fields]) OR (low [All Fields] AND ("health resources"[MeSH Terms] OR "health resources"[All Fields] OR "resources"[All Fields]) AND settings[All Fields]) AND ("loattrfull text"[sb] AND ("2001/01/01"[PDAT] : "2018/09/01"[PDAT]) AND "humans"[MeSH Terms]).

Supplemental Table 1

TRAUMATIC BRAIN INJURY							
**Region**	**Country**	**Author**	**Year**	**Procedure type**	**Type and Size of population**	**Type of study**	**Outcome**
Latin American & Caribbean and North America	Argentina	Pereyra L, et al. (1)	2012	206 patients were subjected to DC. The study did not specify the type of DC.	698 pediatric and adult patients	Retrospective, observational, cross-sectional study in single center	Mortality rate was 48% after DC, and 65% without DC.
		Toledo JA, et al. (2)	2016	42% primary DC, 6% secondary DC. The study did not specify the type of DC.	102 adult patients (>14 years) with head gunshot wounds.	Retrospective observational descriptive study	34% had GOS 4 - 5 points, 24% GOS 2 - 3 points. Mortality was 42%.
	Colombia	Rubiano AM, et al. (3)	2009	Unilateral Early DC in 16 patients compared with non-DC management in 20 patients.	36 pediatric and adult patients	Case control study with a historical control group	Early DC group: 43.7% had GOS 4-5 and 5 patients died. Non-DC group: 35% had GOS 2-3 and 13 patients died.
		Charry JD, et al. (4)	2016	Hemispheric craniectomy	54 adult patients	Retrospective observational study	Forty (74.1%) of the patients survived and 36 (90%) of them had favorable GOS (4-5)
		Charry JD, et al. (5)	2016	Unilateral early DC	106 severe TBI adult patients	Retrospective observational study	74.6% of the patients survived and 66.1% of them had favorable GOS (4-5) at 12 months.
	Cuba	Lacerda AJ, et al. (6)	2013	bi-frontal, unilateral and bilateral DC	12 pediatric patients	Case series	After one year, 58.33% had slight sequelae or no sequelae and 25% died.
		Lacerda AJ, et al. (7)	2018	bi-frontal and unilateral DC	36 pediatric patients	Descriptive study	GOS at 1 year of evolution showed 59.09% with grade V and a mortality of 22.73% (grade I).
	Mexico	Martínez-Bustamante D, et al.(8)	2014	Not specify the type of DC	52 adult patients with penetrating TBI (PTBI)	Retrospective observational study	General mortality was 8%. Mortality in the GCS 3 to 5 points group was 43%, from the 6 to 8 points it was 6%, and no deaths in the GCS 9 to 15 points.
Sub-Saharan Africa	Nigeria	Adeleye AO (9)	2010	Hemispheric DC	17 Adults	Case series	56% of cases with severe head injury had good outcome and the mortality rate in this group was 44%, while 66.7% of the moderate head injury had

							good outcome and the mortality rate was 16.7%.
		Ojo OA et al (10)	2015	Hemispheric DC	10 Adults	Case series	60% of patients after 6 months had GOS 3-5, while 40% died within the first week after DC.
	South Africa	Figaji AA et al(11)	2003	Hemispheric DC	5 pediatric patients	Case series	GOS<br>4-5:100%
		Enslin JN (12)	2011	Hemispheric and bifrontal DC	76 Adults (>14years)	Retrospective observational study	GOS<br>1: 44,4%<br>1-3:66,7%<br>4-5:33,33%
	Cameroon	Motah M, et al.(13)	2014	Bifrontal DC (6) Hemicraniectomy (7)	13 Adults	Case series	10 patients (76.93%), had favorable outcome, 1 remained in vegetative state and 1 died
East Asia Pacific	China	Tang, Z, et al.(14)	2018	Unilateral DC 86.1%, bilateral DC 11.1%, and bifrontal DC 2.8%.	36 pediatric patients	Retrospective observational study	38.9% died within 14 days after DC
		Mao, X, et al(15)	2015	Unilateral and bilateral	207 adults patients	Retrospective observational study	The mortality rate was 39.76% in the DC group, while it was 87.80% in the Conservative Care group
		Bao, Y, et al (16)	2010	Bilateral DC	37 adults patients	Retrospective observational study	54.1% had favorable outcomes and s 45.9% had unfavorable outcomes
		Wang, Z et al (17)	2017	Bifrontal	56 adults patients	Retrospective observational study	58.9% had recovered
		Yu, Pm, et al (18)	2015	unspecified	223 adults patients	Retrospective observational study	Mortality 32.1%. DC reduce the mortality, but failed to improve the long-term prognosis according to the GOS.
		Yuan, Q, et al (19)	2013	Unilateral and bilateral frontal craniectomy	164 pediatric and adults patients (14 – 83	Retrospective cohort study.	22.0% died, 42% had GOS score of 4 to 5 and 36.0% vegetative.
		Wen, L, et al (20)	2011	Unilateral and bilateral frontal craniectomy	44 adults patients	Retrospective observational study	The mortality after 1 month of early DC was 16% compared with 15,8% of mortality after late DC, 6 months mortality 20% of early DC and 21% of late DC
		Qiu, W, et al (21)	2009	unilateral DC and unilateral routine temporoparietal craniectomy	74 adults patients	Prospective randomized clinical trial.	Mortality one month after surgery, was 27% in unilateral DC and 57 in temporoparietal craniectomy. 56.8% of

							patients with good neurological recovery after DC, and 32.4% after temporoparietal craniectomy
		LI, G, et al (22)	2008	large DC and routine decompressive craniectomy	263 pediatric and adults patients	Retrospective observational study	GOS score 3-5 in 71% after large DC and 58.6% after routine decompressive craniectomy
	Malaysia	Sharda P, et al (23)	2014	Unspecified	110 adult and pediatric patients	Retrospective observational study	Mortality of 35%. Post-operative hypoxia, unmaintained CPP and unstable BP were predictors of poor outcomes.
	Mongolia	Wang, Z, et al (24)	2017	Bifrontal	56 adults patients	Retrospective observational study	58.9% had good prognosis, 21.4% had mild disability, 8.9% had severe disability, 5.3% had persistent vegetative status, and5.3% dead.
	Thailand	Limpastan K, et al (25)	2013	Unilateral and Bilateral DC	159 adult and pediatric patients	Retrospective observational study	Mortality after DC was 44.65%. Pre-operative GCS of 6 and higher had a significantly lower mortality when compared with those who scored 5 or lower.
Europe & Central Asia	Turkey	Kalayci M, et al (26)	2013	Unilateral and bilateral DC	34 adult and pediatric patients	Retrospective observational study	After 6 months of DC 47.0% dead and 53.0% survived.
		Cavuşoğlu H, et al (27)	2010	Early unilateral DC	33 adult and pediatric patients	Retrospective observational study	Favorable outcome (mRS 0-3) in 48.5% of patients after one year.
		Ucar, T, et al (28)	2005	Unilateral and bilateral	100 adult and pediatric patients	Retrospective observational study	84% unfavorable outcome and 16% favorable outcome
Middle East, North Africa and South Asia	Iran	Khalili, H., et al. (29)	2017	Unilateral and bilateral	142 adult patients	Retrospective, cross-sectional study	Overall favorable, low GCS on admission and post-operative hydrocephalus associated with unfavorable outcome
	Jordan	Jamous, M., et al. (30)	2010	Bifrontal craniectomy in bilateraly fixed pupils	5 adult patients	Case series	Controlled ICP but all the patients died in 30 days of trauma
	Afghanistan	Ragel, B.T., et al. (31)	2010	Unilateral and bilateral	90 adult patients	Retrospective observational study	Authors recommended L.G.Kempe incision
	Afghanistan and Iraq	Ecker, R. D., et al. (32)	2011	Hemicraniectomy, Bilateral and Bicompartamental	33 Adult patients	Retrospective observational study	Long-term follow-up GOS<br>1: 23%<br>2-3: 17%<br>4-5:60<br>Systemic infections and cardiovascular complications result in worse outcomes
		Bell, R. S., et al. (33)	2010	Unilateral and bilateral	188 Adult patients	Retrospective observational study	Favorable. Authors recommended hemicraniectomy as damage-control before long

							distance transfer
		Roberts, S. A., et al. (34)	2016	Unilateral and bilateral	14 Adult patients	Case series	Favorable. Median GOS 4 (50%) at final follow-up.
	Pakistan	Waqas, M., et al (35)	2018	Unilateral and Bilateral	40 adult patients	Cross-sectional study	Mean GOSE=5.35 +/-1,9 Quality of life after traumatic brain injury (QOLIBRI)=59,65 +/- 21,27
		Khan et al (36)	2018	Unilateral and bilateral	98 adult patients	Retrospective, cross-sectional study	GOSE > 5 in 45.9%. Young age and lower GCS at presentation was associated with worse survival. GCS score on discharge was a strong predictor of functional outcome
		Qasmi et al. (37)	2015	Unilateral	39 adult patients	Observational, cross-sectional study	53.8% had favorable outcome
	India	Prasad, G. L., et al. (38)	2015	Unilateral and bilateral	71 Pediatric	Retrospective observational study	Favorable. Survival advantage in 50% with severe TBI. GOSE: 7–8
		Sinha, S., et al. (39)	2015	Unilateral and bilateral	1236 adult patients	Retrospective observational study	Follow□up of surviving 7.5% persistent vegetative state 78% favorable outcome
		Choudhary NK et al (40)	2018	29%Bifrontal<br>71%Unilateral	85 Adults (male:famale 1,8:1)	Retrospective observational study	GOS: EARLY SURGERY/LATE SURGERY<br>1:10,6%/22,4%<br>2: 12,1%/21,5%<br>3: 16,7%/21,5%<br>4: 28,8%/10,5%<br>5: 31,8%/15,8%

1. PEREYRA C, BENITO MORI L, SCHOON P, et al. Decompressive craniectomy and brain death prevalence and mortality: 8-year retrospective review. *Transplant Proc*. 2012 Sep;44(7):2181-4. doi: 10.1016/j.transproceed.2012.07.107.
2. TOLEDO JA, RE M, PENDINO A, CANULLO ML, et al. Heridos de arma de fuego en cráneo: análisis de 102 casos en población civil en una única institución. *Rev Argent Neuroc*. 2016;30(4):143-9
3. RUBIANO AM, VILLARREAL W, HAKIM EJ, et al. Early decompressive craniectomy for neurotrauma: an institutional experience. *Ulus Travma Acil Cerrahi Derg*. 2009 Jan;15(1):28-38.
4. CHARRY JD, RUBIANO AM, PUYANA JC, et al. Damage control of civilian penetrating brain injuries in environments of low neuro-monitoring resources. *Br J Neurosurg*. 2016;30(2):235-9. doi: 10.3109/02688697.2015.1096905.
5. CHARRY JD, RUBIANO AM, NIKAS CV, ORTIZ JC, PUYANA JC, CARNEY N, ADELSON PD: Results of early cranial decompression as an initial approach for damage control therapy in severe traumatic brain injury in a hospital with limited resources. J Neurosci Rural Pract, 2016, 7(1):7-12
6. LACERDA AJ, PEREZ DA, DIAZ JA, et al. Craniectomía descompresiva en el manejo del traumatismo cráneo–encefálico grave en pediatría. *Rev Cubana Neurol Neurocir*. 2013;3(1):38–43
7. LACERDA AJ, PEREZ DA, PAZOS MM, et al. Decompressive Craniectomy in Pediatric Severe Head Trauma. *Int J Pediatr Res*. 2018;4(1):033. doi.org/10.23937/2469-5769/1510033
8. MARTÍNEZ-BUSTAMANTE D, PÉREZ-CÁRDENAS S, ORTIZ-NIETO JM, et al. Craniocerebral gunshot wounds in civilian population: Analysis of experience in a single center in Monterrey, México. *Cir Cir*. 2015 Mar-Apr;83(2):94-9. doi: 10.1016/j.circir.2015.04.002.
9. ADELEYE AO, Decompressive craniectomy for traumatic brain injury in a developing country: An initial observational study Indian Journal of Neurotrauma (IJNT) 2010, Vol. 7, No. 1, pp. 41-46
10. OJO, O A .BANKOLE, O B, KANU, O O, EGHOSA, M, OLATOSI, O, Early decompressive craniectomy for traumatic brain injury in resource poor centres:a tertiary institution experience. Niger Postgrad Med J 2015 Mar;22(1):45-9.
11. FIGAJI AA, FIEGGEN AG, PETER JC. Early decompressive craniotomy in children with severe traumatic brain injury, Childs Nerv Syst (2003) 19:666–673

12. ENSLIN JM, Outcomes of decompressive craniectomy in adults with severe traumatic brain injury –The Groote Schuur Hospital Experience

13. MOTAH M, UDUMA UF, NDOUMBE A. Surgical Management of Severe Head Injury with Cerebral Herniation. J Neuroinfect Dis. 2014;5(1):136.

14. Tang, Z., Huang, Q., Zhang, J., Yang, R., Wei, W., & Liu, H. (2018). Fourteen-Day Mortality in Pediatric Patients with Traumatic Brain Injury After Early Decompressive Craniectomy: A Single-Center Retrospective Study. World Neurosurgery. doi: 10.1016/j.wneu.2018.07.173

15. Mao, X., Hao, S., Tao, X., Hou, Z., Li, H., & Tian, R. et al. (2015). Decompressive craniectomy for severe traumatic brain injury patients with fixed dilated pupils. Therapeutics And Clinical Risk Management, 1627. doi: 10.2147/tcrm.s89820

16. Bao, Y., Liang, Y., Gao, G., Pan, Y., Luo, Q., & Jiang, J. (2010). Bilateral Decompressive Craniectomy for Patients with Malignant Diffuse Brain Swelling after Severe Traumatic Brain Injury: A 37-Case Study. Journal Of Neurotrauma, 27(2), 341-347. doi: 10.1089/neu.2009.1040

17. Wang, Z., Su, N., Wu, R., Zhang, Y., Zhang, X., & Qi, J. et al. (2017). Decompressive Craniectomy With Bifrontal Coronal Incision in the Management of Fronto-Temporal Contusion and Laceration for Early Cranioplasty. Journal Of Craniofacial Surgery, 28(6), 1442-1444. doi: 10.1097/scs.0000000000003497

18. Yu, P., Tian, Q., Wen, X., Zhang, Z., & Jiang, R. (2015). Analysis of Long-Term Prognosis and Prognostic Predictors in Severe Brain Injury Patients Undergoing Decompressive Craniectomy and Standard Care. Journal of Craniofacial Surgery, 26(7), e635–e641. doi:10.1097/scs.0000000000002063
19. Yuan, Q., Liu, H., Wu, X., Sun, Y., & Hu, J. (2013). Comparative study of decompressive craniectomy in traumatic brain injury with or without mass lesion. British Journal of Neurosurgery, 27(4), 483–488. doi:10.3109/02688697.2013.763897
20. Wen, L., Wang, H., Wang, F., Gong, J. B., Li, G., Huang, X., Yang, X. F. (2011). A prospective study of early versus late craniectomy after traumatic brain injury. Brain Injury, 25(13-14), 1318–1324. doi:10.3109/02699052.2011.608214
21. Qiu, W., Guo, C., Shen, H., Chen, K., Wen, L., & Huang, H. et al. (2009). Effects of unilateral decompressive craniectomy on patients with unilateral acute post-traumatic brain swelling after severe traumatic brain injury. Critical Care, 13(6), R185. doi: 10.1186/cc8178
22. LI, G., WEN, L., YANG, X., ZHENG, X., ZHAN, R., & LIU, W. (2008). Efficacy of large decompressive craniectomy in severe traumatic brain injury. Chinese Journal Of Traumatology (English Edition), 11(4), 253-256. doi: 10.1016/s1008-1275(08)60052-8
23. Sharda P, Haspani S, Idris Z. Factors prognosticating the outcome of decompressive craniectomy in severe traumatic brain injury: A Malaysian experience. Asian J Neurosurg. 2014;9(4):203-12.
24. Wang, Z., Su, N., Wu, R.-L., Zhang, Y.-S., Zhang, X.-J., Qi, J.-J., … Wang, J.-Q. (2017). Decompressive Craniectomy With Bifrontal Coronal Incision in the Management of Fronto-Temporal Contusion and Laceration for Early Cranioplasty. Journal of Craniofacial Surgery, 28(6), 1442–1444. doi:10.1097/scs.0000000000003497
25. Limpastan K, Norasetthada T, Watcharasaksilp W, Vaniyapong T. Factors influencing the outcome of decompressive craniectomy used in the treatment of severe traumatic brain injury. J Med Assoc Thai. 2013;96(6):678-82.
26. Kalayci M, Aktunç E, Gül S, et al. Decompressive craniectomy for acute subdural haematoma: an overview of current prognostic factors and a discussion about some novel prognostic parametres. J Pak Med Assoc. 2013;63(1):38-49.
27. Cavuşoğlu H, Kaya RA, Türkmenoğlu ON, Aydin Y. Value of early unilateral decompressive craniectomy in patients with severe traumatic brain injury. Ulus Travma Acil Cerrahi Derg. 2010;16(2):119-24.
28. Ucar, T., Akyuz, M., Kazan, S., & Tuncer, R. (2005). Role of Decompressive Surgery in the Management of Severe Head Injuries: Prognostic Factors and Patient Selection. Journal of Neurotrauma, 22(11), 1311–1318. doi:10.1089/neu.2005.22.1311.
29. KHALILI, H., NIAKAN, A., GHAFFARPASAND, F., KIANI, A. & BEHJAT, R. 2017. Outcome Determinants of Decompressive Craniectomy in Patients with Traumatic Brain Injury; A Single Center Experience from Southern Iran. *Bull Emerg Trauma,*5**,**190-196.
30. JAMOUS, M., BARBARAWI, M., SAMRAH, S., KHABAZ, M. N., AL-JARRAH, M. & DAUOD, S. 2010. Emergency decompressive craniectomy for trauma patients with Glasgow Coma Scale of 3 and bilateral fixed dilated pupils. *Eur J Trauma Emerg Surg,*36**,**465-9.
31. RAGEL, B. T., KLIMO, P., MARTIN, J. E., TEFF, R. J., BAKKEN, H. E. & ARMONDA, R. A. 2010. Wartime decompressive craniectomy: technique and lessons learned. *Neurosurg Focus,*28**,**E2.
32. ECKER, R. D., MULLIGAN, L. P., DIRKS, M., BELL, R. S., SEVERSON, M. A., HOWARD, R. S. & ARMONDA, R. A. 2011. Outcomes of 33 patients from the wars in Iraq and Afghanistan undergoing bilateral or bicompartmental craniectomy. *J Neurosurg,*115**,**124-9.
33. BELL, R. S., MOSSOP, C. M., DIRKS, M. S., STEPHENS, F. L., MULLIGAN, L., ECKER, R., NEAL, C. J., KUMAR, A., TIGNO, T. & ARMONDA, R. A. 2010. Early decompressive craniectomy for severe penetrating and closed head injury during wartime. *Neurosurg Focus,*28**,**E1.
34. ROBERTS, S. A., TOMAN, E., BELLI, A. & MIDWINTER, M. J. 2016. Decompressive craniectomy and cranioplasty: experience and outcomes in deployed UK military personnel. *Br J Neurosurg,*30**,**529-35.

35. WAQAS, M., MALIK, N., SHAMIM, M. S., NATHANI, K. R. & ABBASI, S. A. 2018. Quality of Life Among Patients Undergoing Decompressive Craniectomy for Traumatic Brain Injury Using Glasgow Outcome Scale Extended and Quality of Life After Brain Injury Scale. *World Neurosurg,*116**,**e783-e790.
36. Khan, F., Valliani, A., Rehman, A., & Bari, M. E. (2018). Factors affecting functional outcome after decompressive craniectomy performed for traumatic brain injury: A retrospective, cross-sectional study. *Asian Journal of Neurosurgery*, *13*(3), 730.
37. Qasmi, S. A., Ghaffar, A., Hussain, Z. & Akram M. 2015. Outcome of descompressive craniectomy (DC)for severe traumatic brain injury (STBI) in adults. Pak Armed Forces Med J 2015; 65(5): 674-78.
38. PRASAD, G. L., GUPTA, D. K., MAHAPATRA, A. K. & SHARMA, B. S. 2015. Surgical results of decompressive craniectomy in very young children: A level one trauma centre experience from India. *Brain Inj,*29**,**1717-24.
39. SINHA, S., RAHEJA, A., GARG, M., MOORTHY, S., AGRAWAL, D., GUPTA, D. K., SATYARTHEE, G. D., SINGH, P. K., BORKAR, S. A., GURJAR, H., TANDON, V., PANDEY, R. M. & SHARMA, B. S. 2015. Decompressive craniectomy in traumatic brain injury: A single-center, multivariate analysis of 1,236 patients at a tertiary care hospital in India. *Neurol India,*63**,**175-83.
40. CHOUDHARY, N. K. & BHARGAVA, R. 2018. Decompressive Craniectomy in Diffuse Traumatic Brain Injury: An Industrial Hospital Study. *Asian J Neurosurg,*13**,**314-318.

Supplemental Table 2

STROKE							
**Region**	**Country**	**Author**	**Year**	**DC Procedure type**	**Type and Size of population**	**Type of study**	**Outcome**
Latin American & Caribbean and North America	Brazil	Vital-Bezzerra R, et al. (1)	2016	Unilateral	60 Adults	Retrospective observational study	mRS<br>5-6: 76% (>60yrs)<br>5-6: 44% (<60yrs)<br>0-4: 24% (>60 yrs)
		Rabelo NN, et al. (2)	2017	Unilateral	37 Adults with aneurysmal subarachnoid hemorrhage or unbroken aneurysms.	Cross-sectional study	Survival 60% (14 asymptomatic, 8 with deficit)<br>Death 40%
	Colombia	Silva F, et al.(3)	2011	Unilateral	18 Adults<br><br>38,8% with DC 61,1% with conventional medical treatment (CMT).	Case series	Mortality<br>DC/ CMT<br>14,2%/ 54,5%
East Asia Pacific	China	Hao, Z, et al (4)	2014	Unilateral	219 adults patients (Of them, 31 patients was DC)	Cohort Study	mRS<br>DC %/NoDC%<br>2: 3.2 / 0.5<br>3:29.0 / 12.8<br>4: 19.4/ 12.8<br>5: 9.7 /12.8<br>6: 38.7/ 61.2
		Shao, A, et al (5)	2013	Unilateral	131 adults (85 witt improve D and 46 with Routine DC)	Comparative, retrospective study	Mortality<br><br>Improve/Routine<br><br>29,41% /63.0 %
		Zhao, J, et al (6)	2012	Large hemicraniectomy	47 adults<br>(24 with DC, 23 with CMT)	Prospective, randomized, controlled trail	DC/CMT<br>Mortality 6 months: 12,5%/60,9%<br><br>Mortality 12 months: 16,7%/69,6%

		Chen, C.-C, et al (7)	2007	Large Decompressive hemicraniectomy	60 adults patients	Retrospective observational study	Survived: 80%<br><br>Mortality<br>- 30 days: 20%<br>- 12 months: 26,6%<br>BI* >60: 65,9%<br><br>*Barthel Index
		Yao, Y, et al (8)	2005	Unilateral DC	25 elderly patients with younger patients.	Retrospective observational study	Mortality<br><60 years: 7,7%<br>>60 years: 33,3%.
		Yang, X, et al (9)	2005	Unilateral DC	24 adults patients	Retrospective observational study	Mortality was 10.0% after DC compared with 64.2% after medical treatment alone
		Cho, D, et al (10)	2003	Unilateral DC	52 adults patients	Retrospective observational study	8.3% of mortality at 30 days after ultra-early DC, 36.7% after DC beyond 6 hours, and 80% no operation.
		Zhao, B, et al (11)	2015	Primary Unilateral DC	24 adults patients with MCA aneurysm with associated intracerebral hemorrhage	Multicenter retrospective case-control study	58% patients had a favorable outcome and the mortality was 29%
	Malaysia	Raffiq, M, et al (12)	2014	Unilateral DC	125 adults patients	Retrospective observational study	Mortality was 30.0% after DC and 54.3% after medical treatment, favorable outcome was in 37.8% after DC and 2.9% after medical treatment at 30 days
Europe & Central Asia	Turkey	Gulensoy, B, et al.(13)	2014	Decompressive hemicraniectomy	42 adults patients	Retrospective observational study	27 cases with unfavorable outcome and 15 with favorable outcome.
		Kilincer, C., et al (14)	2005	Unilateral	32 adults patients	Non-randomized prospective study	Mortality after one month 31% and most of the surviving patients were severely disabled (RS 4 or 5)
Middle East, North Africa and South Asia	Iran	Rahmanian, A, et al. (15)	2014	Hemicraniectomy	60 adult patients	Case-control study	Mortality was 20% after DC and 67% after medical treatment
		Rasras, S, et al. (16)	2018	Hemicraniectomy without clot evacuation	30 adult patients	Randomized clinical trial	DC without clot evacuation in deep seated ICH can be accomplished with identical mortality and outcome in comparison to patient that undergone clot evacuation. Mortality was 30.8% in case and 29.4% in control

	Egypt	Moussa, W. M, et al. (17)	2017	Hemicraniectomy with expansile duraplasty	40 adult patients (DC and non-DC)	Randomized clinical trial	At 6 months' follow-up, 14 (70 %) patients of group A (DC) had favorable outcome as compared to 4 (20 %) patients of group B (non-DC)
	Pakistan	Kamal Alam, B, et al. (18)	2017	Hemicraniectomy	34 adult patients	Longitudnal cohort study	Mild improvements in functional outcomes after six months of malignant MCA infarcts but with poor functional outcomes as a whole (mRS > 4 and mBI < 60).
		Khatri, I. A, et al. (19)	2008	Hemicraniectomy	5 adult patients	Case series	Not mortility<br>Complication:<br>60%: aspiration pneumonia.<br>40% septicaemia in 20% seizures and status epilepticus.
		Khan et al (20)	2010	Hemicraniectomy (occipital) for cerebellar stroke	6 adult patients	Case series	GOS<br>5： 83,4 %<br>1: 16,6%
	India	Rai, V. K, et al.(21)	2014	Hemicraniectomy	60 adult patients	Prospective observational study	Favorable. Reduced mortality and disability. Mortality at 1 year was 38% in DC and 83% in Medical treatment
		Bansal, H, et al. (22)	2015	Hemicraniectomy	53 adult patients	Retrospective observational study	DC has reduced morbidity and mortality especially in people aged below 60 years and those operated within 48 h of malignant MCA stroke
		Kumar, A, et al. (23)	2013	Hemicraniectomy	18 adult patients	Retrospective observational study	Favorable. Survival rates were 62.5% at 1 year
		Ramnarayan, R, et al. (24)	2009	Hemicraniectomy	23 adult patients	Retrospective observational study	Favorable in 13, Unfavorable in 10(including 3 deaths)
		Pranesh, M. B, et al.(25)	2003	Hemicraniectomy	19 adult patients	Case series	The functional outcome is good in younger patients
		Pillai, A, et al.(26)	2007	Hemicraniectomy	26 adult patients	Non-randomized prospective study	Favorable.73% survival at 1-year follow up. Among survivors 72% were independent at 1-year follow up
**Cerebral Venous Sinus Thrombosis (CVST)**							
East Asia Pacific	China	Zhang, S, et al (27)	2017	Unilateral	58 adults patients	Retrospective observational study	56.9% favorable outcome, 13,8% died
Middle East, North Africa and South Asia	Pakistan	Raza, E, et al. (28)	2014	Hemicraniectomy	7 adult patients	Case series	Favorable in those who had reactive pupils pre-operatively
	India	Rajan Vivakaran, T. T, et al. (29)	2014	Hemicraniectomy	34 adult patients	Retrospective observational study	Favorable. 26 (76.4%) had GOS 4 or 5
		Aaron, S, et al. (30)	2013	Unilateral and bilateral	44 adult patients	Retrospective observational study	Favorable, Mortality was 9 (20%)
		Mohindra, S, et al. (31)	2011	Hemicraniectomy	13 adult patients	Retrospective observational study	Favorable. 11 (84.6%) survived with GOS 4 or 5
		Lath, R, et al. (32)	2010	Hemicraniectomy	11 adult patients	Retrospective observational study	Favorable. 73% survival and died 27%

1. VITAL RB, HAMAMOTO FILHO PT, LUVIZUTTO GJ, et al. Decompressive Hemicraniectomy in a South American Population--Morbidity and Outcomes Analysis. *PLoS One*. 2016 Jan 14;11(1):e0146747. doi: 10.1371/journal.pone.0146747.
2. RABELO NN, FERRARI MA, RABELO NN et al. Descompressive Craniectomy in Aneurysms surgery. When and how to do it?. Rev. *Chil. Neurocirugía*. 2017;43(1):150-155
3. SILVA F, SALGADO SA, RUIZ N, et al. Hemicranectomia en pacientes con ataque cerebro vascular isquémico extenso: aspectos relevantes a propósito de una serie de casos. *Acta Neurol Colomb*. 2011;27(1):137-145
4. HAO, Z., CHANG, X., ZHOU, H., LIN, S., & LIU, M. (2015). A Cohort Study of Decompressive Craniectomy for Malignant Middle Cerebral Artery Infarction. Medicine, 94(25), e1039.
5. SHAO, A., GUO, S., CHEN, S., SUN, C., WU, H., HONG, Y., & ZHANG, J. (2013). Comparison Between Routine and Improved Decompressive Craniectomy on Patients With Malignant Cerebral Artery Infarction Without Traumatic Brain Injury. Journal of Craniofacial Surgery, 24(6), 2085–2088.
6. ZHAO, J., SU, Y. Y., ZHANG, Y., ZHANG, Y. Z., ZHAO, R., WANG, L., GAO, D. (2012). Decompressive Hemicraniectomy in Malignant Middle Cerebral Artery Infarct: A Randomized Controlled Trial Enrolling Patients up to 80 Years Old. Neurocritical Care, 17(2), 161–171.
7. CHEN, C.-C., CHO, D.-Y., & TSAI, S.-C. (2007). Outcome and Prognostic Factors of Decompressive Hemicraniectomy in Malignant Middle Cerebral Artery Infarction. Journal of the Chinese Medical Association, 70(2), 56–60.
8. YAO, Y., LIU, W., YANG, X., HU, W., & LI, G. (2005). Is decompressive craniectomy for malignant middle cerebral artery territory infarction of any benefit for elderly patients? Surgical Neurology, 64(2),
9. YANG, X., YAO, Y., HU, W., LI, G., XU, J., ZHAO, X., & LIU, W. (2005). Is decompressive craniectomy for malignant middle cerebral artery infarction of any worth?. Journal Of Zhejiang University SCIENCE, 6B(7), 644-649.
10. CHO, D., CHEN, T., & LEE, H. (2003). Ultra-early decompressive craniectomy for malignant middle cerebral artery infarction. Surgical Neurology, 60(3), 227-232.
11. ZHAO, B., ZHAO, Y., TAN, X., CAO, Y., WU, J., ZHONG, M., & WANG, S. (2015). Primary decompressive craniectomy for poor-grade middle cerebral artery aneurysms with associated intracerebral hemorrhage. Clinical Neurology and Neurosurgery, 133, 1–5.
12. RAFFIQ, M. A., HASPANI, M. S., KANDASAMY, R., & ABDULLAH, J. (2014). Decompressive craniectomy for malignant middle cerebral artery infarction: Impact on mortality and functional outcome. Surgical Neurology International, 5(1), 102.
13. GULENSOY, B., KARATAY, M., ERDEM, Y., CELIK, H., TASCIOGLU, T., & SERTBAS, I. et al. (2014). Decompressive hemicraniectomy for malignant middle cerebral artery infarct. Turkish Neurosurgery.
14. KILINCER, C., ASIL, T., UTKU, U., HAMAMCIOGLU, M. K., TURGUT, N., HICDONMEZ, T. COBANOGLU, S. (2005). Factors affecting the outcome of decompressive craniectomy for large hemispheric infarctions: a prospective cohort study. Acta Neurochirurgica, 147(6), 587–594
15. RAHMANIAN, A., SEIFZADEH, B., RAZMKON, A., PETRAMFAR, P., KIVELEV, J., ALIBAI, E. A. & HERNESNIEMI, J. (2014). Outcome of decompressive craniectomy in comparison to nonsurgical treatment in patients with malignant MCA infarction. *Springerplus,*3**,**115.
16. RASRAS, S., SAFARI, H., ZEINALI, M. & JAHANGIRI, M. (2018). Decompressive hemicraniectomy without clot evacuation in supratentorial deep-seated intracerebral hemorrhage. *Clin Neurol Neurosurg,*174**,**1-6.
17. MOUSSA, W. M. & KHEDR, W. (2017). Decompressive craniectomy and expansive duraplasty with evacuation of hypertensive intracerebral hematoma, a randomized controlled trial. *Neurosurg Rev,*40**,**115-127.
18. KAMAL ALAM, B., BUKHARI, A. S., ASSAD, S., MUHAMMAD SIDDIQUE, P., GHAZANFAR, H., NIAZ, M. J., KUNDI, M., SHAH, S. & SIDDIQUI, M. (2017). Functional Outcome After Decompressive Craniectomy in Patients with Dominant or Non-Dominant Malignant Middle Cerebral Infarcts. *Cureus,*9**,**e997.
19. KHATRI, I. A., SIDDIQUI, M., KHAN, I., NADEEM, M. & AHMAD, A. (2008). Salvage decompressive craniectomy in malignant MCA infarcts--results of local experience at Shifa International Hospital, Islamabad, Pakistan. *J Pak Med Assoc,*58**,**404-8.
20. KHAN, I., DIN JANJUA, M. B. U., KHATRI, I. A., & NADEEM, M. (2010). Surgical decompression in massive cerebellar stroke. *Rawal Medical Journal*, *35*(1), 96-100.
21. RAI, V. K., BHATIA, R., PRASAD, K., PADMA SRIVASTAVA, M. V., SINGH, S., RAI, N. & SURI, A. (2014). Long-term outcome of decompressive hemicraniectomy in patients with malignant middle cerebral artery infarction: a prospective observational study. *Neurol India,*62**,**26-31.
22. BANSAL, H., CHAUDHARY, A., SINGH, A., PAUL, B. & GARG, R. (2015). Decompressive craniectomy in malignant middle cerebral artery infarct: An institutional experience. *Asian J Neurosurg,*10**,**203-6.
23. KUMAR, A., SHARMA, M. S., SHARMA, B. S., BHATIA, R., SINGH, M., GARG, A., KUMAR, R., SURI, A., CHANDRA, P. S., KALE, S. S. & MAHAPATRA, A. K. (2013). Outcome after decompressive craniectomy in patients with dominant middle cerebral artery infarction: A preliminary report. *Ann Indian Acad Neurol,*16**,**509-15.
24. RAMNARAYAN, R., ANTO, D., ANILKUMAR, T. V. & NAYAR, R. (2009). Decompressive hemicraniectomy in large putaminal hematomas: an Indian experience. *J Stroke Cerebrovasc Dis,*18**,**1-10.
25. PRANESH, M. B., DINESH NAYAK, S., MATHEW, V., PRAKASH, B., NATARAJAN, M., RAJMOHAN, V., MURALI, R. & PEHLAJ, A. (2003). Hemicraniectomy for large middle cerebral artery territory infarction: outcome in 19 patients. *J Neurol Neurosurg Psychiatry,*74**,**800-2.
26. PILLAI, A., MENON, S. K., KUMAR, S., RAJEEV, K., KUMAR, A. & PANIKAR, D. (2007). Decompressive hemicraniectomy in malignant middle cerebral artery infarction: an analysis of long-term outcome and factors in patient selection. *J Neurosurg,*106**,**59-65.
27. ZHANG, S., ZHAO, H., LI, H., YOU, C., & HUI, X. (2017). Decompressive craniectomy in hemorrhagic cerebral venous thrombosis: clinicoradiological features and risk factors. Journal Of Neurosurgery, 127(4), 709-715. doi: 10.3171/2016.8.jns161112

28. RAZA, E., SHAMIM, M. S., WADIWALA, M. F., AHMED, B. & KAMAL, A. K. (2014). Decompressive surgery for malignant cerebral venous sinus thrombosis: a retrospective case series from Pakistan and comparative literature review. *J Stroke Cerebrovasc Dis,*23**,**e13-22.
29. RAJAN VIVAKARAN, T. T., SRINIVAS, D., KULKARNI, G. B. & SOMANNA, S. (2012). The role of decompressive craniectomy in cerebral venous sinus thrombosis. *J Neurosurg,*117**,**738-44.
30. AARON, S., ALEXANDER, M., MOORTHY, R. K., MANI, S., MATHEW, V., PATIL, A. K., SIVADASAN, A., NAIR, S., JOSEPH, M., THOMAS, M., PRABHU, K., JOSEPH, B. V., RAJSHEKHAR, V. & CHACKO, A. G. (2013). Decompressive craniectomy in cerebral venous thrombosis: a single centre experience. *J Neurol Neurosurg Psychiatry,*84**,**995-1000.
31. MOHINDRA, S., UMREDKAR, A., SINGLA, N., BAL, A. & GUPTA, S. K. (2011). Decompressive craniectomy for malignant cerebral oedema of cortical venous thrombosis: an analysis of 13 patients. *Br J Neurosurg,*25**,**422-9.
32. LATH, R., KUMAR, S., REDDY, R., BOOLA, G. R., RAY, A., PRABHAKAR, S. & RANJAN, A. (2010). Decompressive surgery for severe cerebral venous sinus thrombosis. *Neurol India,*58**,**392-7.

Supplemental Table 3

OTHER PATHOLOGIES								
**Region**	**Country**	**Author**	**Pathology**	**Year**	**Procedure type**	**Type and Size of population**	**Type of study**	**Outcome**
Latin America and Caribean Region	Argentina	Montaña S, et al. (1)	Infection	2017	Unilateral DC	A. schindleri (As190) blaNDM-1-positive strain isolated in a 52-year-old woman with a history of positive serology for HIV and hepatitis C virus.	Case report	After being treated with ceftazidime for 4 weeks and then discharged with good clinical evolution.
	Brazil	Oliveira FA, et al. (2)	Infection	2018	Posterior fossa decompressive craniectomy	A 8 year old boy who presented with Chiari I malformation and recurrent meningitis without fistula	Case report	The patient died due to destruction of the cervical skull transition and consequent medullary bulb compression
	Peru	Rojas-Jaimes J, et al. (3)	Infection	2017	Unilateral DC	Pharyngitis in a 12-year-old girl which later developed into a subdural empyema caused by the bacteria Peptostreptococcus sp	Case report	Seven months after hospital discharge the patient received cranioplasty with miniplates to restore the skullcap
	Argentina	Iaconis J, et al. (4)	Tumor	2018	Unilateral DC	A 5 year old girl	Case report	The patient passed away six months later.
	Mexico	Monroy-Sosa A, et al. (5)	Tumor	2018	Unilateral DC	A 34-year-old male with brain angiometastasis from a non-seminomatous germ cell tumor	Case report	The patient showed good recovery in terms of his overall status in the first month after surgery, but the remaining brain metastases grew and bled with rostrocaudal deterioration and death.
Middle East, North Africa and South Asia	India	Singhi, P et al (6)	Infection	2014	Hemicraniectomy	1 pediatric patient	Case report	At discharge, he had normal sensorium and memory and recovering aphasia, residual right hemiparesis, ptosis, and asymmetry of lower half of the face.

		Agarwal et al (7)	Infection	2005	Hemicraniectomy	1 adult patient	Case report	The patient improved remarkably following surgery. He became conscious and his Glasgow Coma Score improved to 14.
Europe & Central Asia	Turkey	Tuzgen S, et al (8)	Vasospasm after aneurysmal subarachnoid hemorrhage	2012	Unilateral	6 adults patients	Case series	After one year, 1 patient died, 2 patients had moderate disability (mRS of 4) and 3 patients continue their life with minimal deficit and no major dependency.
Middle East, North Africa and South Asia	Bangladesh	Rahman, M, et al. (9)	TBI, MCA infarct, ICH, ASDH	2017	Unilateral	32 adult patients	Cross-sectional observational study	Improves survival but quality of life limited especially in poor GCS income (3-6) group.
	India	Mathai et al (10)	Increased ICP(TBI, MCA infarct, CVST)	2008	Hemicraniectomy	12 adult patients	Cohort study	4 patients in this cohort died, while 5 had good outcomes
		Salunke et al. (11)	Large demyelinating lesions	2012	Hemicraniectomy	8 adult patients	Case series	Favorable, 3 from 8 patient died
		Singh et al. (12)	Intracerebral hemorrhage in acute leukemia	2017	Hemicraniectomy	2 pediatric patients with acute leukemia	Case report	In both patients the neurological status improved after DC with hemiparesis, which improved gradually.

1. MONTAÑA, S., PALOMBARANI, S., CARULLA, M., et al. (2018) First case of bacteraemia due toAcinetobacter schindleriharbouring blaNDM-1in an immunocompromised patient. *New Microbe and New Infect*. 21:28–30. (ARGENTINA)
2. OLIVEIRA, FA., MOREIRA FABIÃO, O., GIBBON, FL., et al. (2018). Gorham's Syndrome Associated with Chiari I Malformation and Recurrent Meningitis without Fistula: Case Report. *Journal of Clinical and Diagnostic Research*. 12(5): PD17-PD19. doi: 10.7860/JCDR/2018/35304.11565
3. ROJAS-JAIMES, J., DIAZ-TELLO. A,, CARPIO BAZÁN, C.. (2017). Subdural empyema caused by Peptostreptococcus sp.: a complication of acute pharyngitis. *Rev Inst Med Trop São Paulo*. 59:e83.
4. IACONIS, J., COPPOLA, F., VOLPE, E., et al. (2018). Hemorrhagic Presentation of Isolated CNS Neuroblastoma Recurrence: Case Report. *EC Neurology*. 10(9):848-851. (ARGENTINA)
5. MONROY-SOSA, A., REYES-SOTO, G., CACHO-DÍAZ, B. (2018) Brain angiometastasis from a non-seminomatous germ cell tumor: A case report. *Int J Surg Case Rep*. 42:44-49. doi: 10.1016/j.ijscr.2017.11.053
6. SINGHI, P., SAINI, A. G., SAHU, J. K., KUMAR, N., VYAS, S., VASISHTA, R. K. & AGGARWAL, A. (2015). Unusual Clinical Presentation and Role of Decompressive Craniectomy in Herpes Simplex Encephalitis. *J Child Neurol,*30**,**1204-7.
7. AGRAWAL, D. & HUSSAIN, N. (2005). Decompressive craniectomy in cerebral toxoplasmosis. *Eur J Clin Microbiol Infect Dis,*24**,**772-3.
8. TUZGEN, S., ABUZAYED, B., KUCUKYURUK, B., OZLEN, F., KIZILKILIC, O., & AYDIN, S. (2012). Decompressive craniectomy in patients with cerebral infarction due to malignant vasospasm after aneurysmal subarachnoid hemorrhage. *Journal Of Neurosciences In Rural Practice*, 3(3), 251. doi: 10.4103/0976-3147.102598
9. RAHMAN, M. M., UDDIN, K. H., HARUN, K., BARUA, K. K., KHAN, A. M., & SALAM, M. A. (2017). Surgical Outcome of Decompressive Craniectomy: Study of 32 Cases. *Journal of National Institute of Neurosciences Bangladesh*, *3*(2), 80-83.
10. MATHAI, K. I. & SAHOO, P. K. 2008. Decompressive craniectomy: an effective but underutilized option for intracranial pressure management. *Indian J Surg,*70**,**181-3.
11. SALUNKE, P., AGGARWAL, A., GUPTA, K., AGRAWAL, P., AHUJA, C. K. & VASISHTA, R. K. 2012. Large demyelinating lesions: a neurosurgical perspective. *Br J Neurosurg,*26**,**490-8.
12. SINGH, J., GARG, K., SHARMA, R., SINHA, S. & KALE, S. S. 2017. Damage control surgery in intracerebral hemorrhage in acute leukemia: a review of two cases. *Childs Nerv Syst,*33**,**1229-1232.
